# Environmental Stressors Modulating Seasonal and Daily Carbon Dioxide Assimilation and Productivity in *Lessonia spicata*

**DOI:** 10.3390/plants14152341

**Published:** 2025-07-29

**Authors:** Macarena Troncoso, Zoë L. Fleming, Félix L. Figueroa, Nathalie Korbee, Ronald Durán, Camilo Navarrete, Cecilia Rivera, Paula S. M. Celis-Plá

**Affiliations:** 1 Doctorado Interdisciplinario en Ciencias Ambientales, Facultad de Ciencias Naturales y Exactas, Universidad de Playa Ancha, Valparaíso 2360004, Chile; macarena.troncoso@upla.cl (M.T.); camilo.navarrete@upla.cl (C.N.); 2 Doctorado en Biotecnología Avanzada, Facultad de Ciencias, University of Malaga, 29010 Malaga, Spain; 3 Laboratorio de Investigación Ambiental Costero/HUB Ambiental UPLA, Facultad de Ciencias Naturales y Exactas, Universidad de Playa Ancha, Valparaíso 2360004, Chile; 4 Centro de Investigación en Tecnologías para la Sociedad, Facultad de Ingeniería, Universidad del Desarrollo, Santiago 7610685, Chile; zfleming@udd.cl; 5 Institute of Blue Biotechnology and Development (IBYDA), University of Malaga, 29071 Malaga, Spain; felix_lopez@uma.es (F.L.F.); nkorbee@uma.es (N.K.); 6 Departamento de Filosofía, Historia y Turismo, Facultad de Humanidades, Universidad de Playa Ancha, Valparaíso 2370957, Chile; ronald.duran@upla.cl; 7 Laboratorio de Química Ambiental (Lab QA), Departamento de Ciencias y Geografía, Facultad de Ciencias Naturales y Exactas, Universidad de Playa Ancha, Valparaíso 2360004, Chile; cecilia.rivera@upla.cl

**Keywords:** carbon assimilation, environmental stressors, *Lessonia spicata*, photosynthetic activity, productivity

## Abstract

Carbon dioxide (CO_2_) emissions due to human activities are responsible for approximately 80% of the drivers of global warming, resulting in a 1.1 °C increase above pre-industrial temperatures. This study quantified the CO_2_ assimilation and productivity of the brown macroalgae *Lessonia spicata* in the central Pacific coast of Chile, across seasonal and daily cycles, under different environmental stressors, such as temperature and solar irradiance. Measurements were performed using an infra-red gas analysis (IRGA) instrument which had a chamber allowing for precise quantification of CO_2_ concentrations; additional photophysiological and biochemical responses were also measured. CO_2_ assimilation, along with the productivity and biosynthesis of proteins and lipids, increased during the spring, coinciding with moderate temperatures (~14 °C) and high photosynthetically active radiation (PAR). Furthermore, the increased production of photoprotective and antioxidant compounds, including phenolic compounds, and carotenoids, along with the enhancement of non-photochemical quenching (NPQ), contribute to the effective photoacclimation strategies of *L. spicata*. Principal component analysis (PCA) revealed seasonal associations between productivity, reactive oxygen species (ROSs), and biochemical indicators, particularly during the spring and summer. These associations, further supported by Pearson correlation analyses, suggest a high but seasonally constrained photoacclimation capacity. In contrast, the reduced productivity and photoprotection observed in the summer suggest increased physiological vulnerability to heat and light stress. Overall, our findings position *L. spicata* as a promising nature-based solution for climate change mitigation.

## 1. Introduction

The recent changes to the climate, due to rising anthropogenic greenhouse gas (GHG) emissions, particularly CO_2_, have been unprecedented across hundreds of thousands of years [1]. Nature-based solutions (NbS) like marine vegetated habitats play a key role in climate change mitigation by enhancing carbon storage and sequestration [2,3,4]. The assimilation of inorganic carbon (C) by macroalgae through photosynthesis is the most important energy base of highly productive coastal ecosystems [5,6]. Indeed, this assimilation is conducted by capturing atmospheric CO_2_ and/or its dissolved inorganic form (DIC) in water as a bicarbonate (HCO_3_^−^), under certain temperature and light conditions [7,8,9,10]. Once inorganic C is assimilated and subsequently fixed, it can be sequestered in its organic form through four main pathways: released as dissolved organic carbon (DOC) and able to be exported to other environments (horizontal transport); released as particulate organic carbon (POC), which can be buried in marine sediments (vertical transport); exported from DOC and/or POC to deep-water areas with long residence times; used as a biomass for primary consumers (C donor) due to its high content of macronutrients as reserve carbohydrates (up to 32–60% of dry weight), followed by proteins (7–31% of dry weight) and lipids (2–13% of dry weight), which differ according to the species, geographic location, seasonal cycle and environmental conditions [9,11,12,13,14,15,16].

Climate change exposes rocky intertidal macroalgae to environmental stressors, such as temperature, PAR (photosynthetically active radiation) and high doses of UV (ultraviolet radiation) with negative effects on their growth, reproduction, diversity, biosynthesis of macronutrients and physiology [11,17,18,19]. Indeed, the macroalgae in the coastal zones, exhibit high carbon-to-nitrogen (C: N) ratios and achieve net primary productivity (NPP) rates of ~1826 Tg C yr^−1^ that correspond to ~1% of global primary productivity with ~199 Tg C effectively sequestered [3,20,21,22]. In this sense, the excess of irradiance and elevated temperatures has a negative effect on pigment compositions such as chlorophylls and carotenoids, leading to PSII photoinhibition, electron transport chain disruption, and reduced ATP and NADPH production, which are essential for CO_2_ fixation [23,24]. Furthermore, several studies have demonstrated that global warming and marine heatwaves significantly impact the phenology and physiology of brown macroalgae, affecting their growth, size, and overall performance at both early and adult stages [25,26,27,28]. Wright et al. [29] analyzed how shifts in the composition of brown seaweed forests due to ocean warming could alter their carbon sequestration potential. Species such as *L. digitata* and *L. hyperborea* are being replaced by the warm-adapted *L. ochroleuca*. While *L. ochroleuca* exports up to 71% more carbon than the cold-water species, its organic matter decomposes 155% faster, reducing its long-term carbon sequestration efficiency. These findings highlight the need for further research to establish the optimal thermal thresholds for both carbon sequestration and organic matter decomposition in other brown macroalgae. Consequently, the high levels of UV radiation exacerbate stress by generating reactive oxygen species (ROSs), causing oxidative damage to cellular structures [19,30]. To counteract these effects, intertidal macroalgae adopt protective strategies, including synthesizing photoprotective and antioxidant compounds, morphological adaptations, and physiological plasticity [19,31,32,33,34]. Understanding their physiological and biochemical responses is therefore crucial to assessing the impact of these stressors on CO_2_ assimilation, productivity, and biosynthesis of macronutrients in macroalgae [6,11,19,23].

In the central coast of Chile (29–36° S), understanding the physiological, photochemical, and biochemical responses of the *L. spicata* macroalgae to environmental variability is critical for assessing its role in carbon dynamics under climate change scenarios [19,35,36]. This study is the first to evaluate the seasonal and daily variation in CO_2_ assimilation, primary productivity, and macronutrient biosynthesis in *L. spicata*, aiming to elucidate the influence of environmental stressors as temperature and solar irradiance (PAR/UVA) on their photoacclimation capacity. We hypothesize that *L. spicata* exhibits higher CO_2_ assimilation productivity and macronutrient biosynthesis during the central hours of the day in the spring and summer time, when temperature and irradiance are elevated. The findings will enhance our understanding of *L. spicata* as a carbon assimilator and open the discussion that seaweed might be considered as mitigators of the climate change scenarios in the Pacific Ocean.

## 2. Material and Methods

### 2.1. Sampling and Study Area

**Figure 1:**
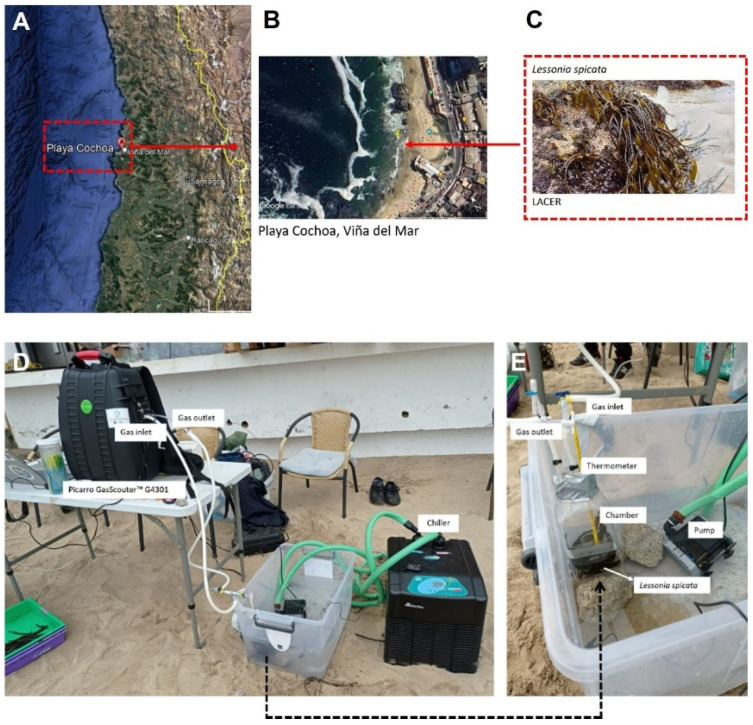
(**A**) Location of the sampling site: Playa Cochoa, Viña del Mar, Valparaíso region (32°57′S, 71°32′W). (**B**) Scheme of the in-situ experiment for the quantification of the CO_2_ assimilation of *L. spicata*. (**C**) Specie of study. (**D**) Set up experimental CO_2_ assimilation with IRGA and cambers with temperature control. (**E**) Details of the chamber to measure CO_2_ assimilation.

The brown seaweed *L. spicata* (Surh) Santelices (Phaeophyceae, Laminariales) is the most abundant species found along the Chilean coast and is distributed from central Chile (29° S) to the south of the Gulf of Penas (46°59′–47°40′ S) [37,38]. Six fully grown *L. spicata* samples were randomly collected in an intertidal zone at a minimum distance of 10 m from the shore along a 70 m transect in Playa Cochoa (Figure 1A), Valparaíso region (32°57′19” S; 71°32′52″ W), during each season in 2022 (summer, autumn, winter, and spring) along a daily cycle (at 10:00, 14:00 and 18:00 h, according to Local Time). In situ physiological measurements were then made in a container with 1.5 L of seawater. At the same time, thallus samples from *L. spicata* were frozen in liquid nitrogen and transported to the Laboratory of Costal Environmental Research at the Environmental HUB, Universidad de Playa Ancha, and preserved at −80 °C for biochemical analysis.

Seawater temperature, salinity, pH, and conductivity were measured using a multiparameter water quality meter (HI 98194, Hanna Instruments, Woonsocket, RI, USA). Changes in the spectral composition of solar radiation, PAR (λ = 400–700 nm), and ultraviolet-A and -B (UVA) radiation (λ = 315–400 nm) were quantified through Apogee sensors (Apogee Instruments, Logan, UT, USA) with a data logger (HOBO UX120-006M, Onset Computer Corporation, Bourne, MA, USA) according to the method detailed by Celis-Plá et al. [19].

### 2.2. Photosynthetic Activity

#### 2.2.1. CO_2_ Assimilation and Quantification

CO_2_ concentration (ppm) was measured in a closed equilibration chamber containing 15 g of fresh weight (FW) *L. spicata* and 500 mL of seawater over a 1 h incubation period. Gas-phase CO_2_ levels were monitored in real time using a GasScouter G4301 (Picarro, Santa Clara, CA, USA) infrared spectrometer, which operates via cavity-ring-down spectroscopy (CRDS) [39,40]. This technique uses a three-mirror optical cavity to maintain a continuous infrared light path, enhancing detection sensitivity and measurement precision. Once equilibrium was reached (Figure 1B), the amount of assimilated carbon (mg C–CO_2_/kg) was calculated following the approach described by Stock et al. [41].

In the first step, the number of CO_2_ moles (nCO_2_) present in the chamber was determined using the ideal gas law (Equation (1)).

(1) $$nCO2=\frac{P\times V}{R\times T}$$

where *P* is the partial pressure of CO_2_ (Pa), V is the volume of CO_2_ in the equilibrium chamber (m^3^), R is the ideal gas constant (8.314 m^3^ Pa/mol K), and T is the system temperature (K).

In the second step, the nCO_2_ value was then converted into carbon mass based on the chamber volume and algal biomass, as described according to Equation (2).

(2) $${Mass}_{C-CO2\;(chamber)}=n_{CO2}\times {MM}_{CO2}\times 1000$$

where *MM_CO2_* is the molar mass of CO_2_ (44.01 g mol^−1^).

Finally, CO_2_ assimilation was normalized by the algal biomass of *L. spicata* and was calculated according to Equation (3):

(3) $${Mass}_{C-CO2\;/kg\;FW}=\frac{{Mass}_{C-CO2\;(chamber)}}{Biomass\left(L.\;spicata\right)\;in\;kg}\times 1000$$

where biomass refers to the fresh weight of the algal sample (15 g). The net CO_2_ assimilation rate was expressed in units of mg C kg^−1^ FW h^−1^, indicating the amount of carbon assimilated per kilogram of fresh biomass per hour.

#### 2.2.2. *In Vivo* Chlorophyll *a* Fluorescence

In Vivo fluorescence of chlorophyll-*a* (chl*a*) associated with photosystem II (PSII) as an estimator of photosynthetic activity was determined using a Mini PAM II portable fluorometer with WinControl-3.2 software (Walz GmbH, Effeltrich, Germany). *L. spicata* samples were placed in 10 mL incubation chambers with seawater to obtain Rapid Light Curves (RLCs) during the daily cycle. The RLCs represent the saturation characteristics of PSII electron transport and overall photosynthetic performance [42].

#### 2.2.3. Maximum Quantum Yield (F_v_/F_m_)

To determine the maximum quantum yield (*F_v_*/*F_m_*), algal thalli were incubated in a dark chamber with fresh seawater for 15 min before measuring the RLCs to completely photo-reduce all reaction centers [19]. Once the *L. spicata* samples were in a dark-adapted state, the minimum (*F_o_*) and maximum (*F_m_*) quantum yields were determined. The maximum quantum yield of PSII (*F_v_*/*F_m_*) derived from the parameters *F_m_* and *F_o_*, according to Schreiber et al. [43], is an estimator of the physiological state and photoinhibition of the thalli:

(4) $$\frac{F_{v}}{F_{m}}=\frac{\left(F_{m}^{\prime }-F_{0}\right)}{F_{m}^{\prime }}$$

where *F′_m_* is the maximum fluorescence induced with a pulse of saturating white light and *F_o_* is the current steady-state fluorescence in light-adapted algae.

#### 2.2.4. Electron Transport Rate (ETR)

The electron transport rate (ETR) is an indicator of photosynthetic productivity and capacity, and was determined by exposing the tissue for a period of 12 incremental irradiances of actinic light: E1 = 25, E2 = 45, E3 = 66, E4 = 90, E5 = 125, E6 = 190, E7 = 285, E8 = 420, E9= 625, E10= 845, E11= 1150 and E12= 1500 μmol photons m^2^ s^−1^; this was carried out according to the methodology detailed by Celis-Plá et al. [19]. The ETR was calculated according to Equation (5) [43]:

(5) $$ETR\left(µmol\;electron\;m^{-2}\;s^{-1}\right)=\frac{\Delta F}{F_{m}^{\prime }}\times E\times A\times FII$$

where the *ΔF*/*F′_m_* is the effective quantum yield; E is the incident PAR irradiance expressed in μmol photons m^−2^ s^−1^; A is the thallus absorptance equivalent to the fraction of incident irradiance that is absorbed by algae estimated using a PAR sensor [44]; and FII is the fraction of Chl*a* associated with PSII (400-700 nm), which is 0.8 in brown macroalgae such as *L. spicata*, according to Figueroa et al. [45]. Photosynthetic parameters such as the maximum electron transport rate (ETR*_max_*) and photosynthetic efficiency (α_ETR_) were calculated from the tangential model reported by Eilers and Peeters [46].

##### Non-Photochemical Quenching (NPQ)

*NPQ* is one of the main photoprotection mechanisms of photosynthetic organisms, according to Celis-Plá et al. [42] (Equation (6)):

(6) $$NPQ=\frac{F_{m}/F_{m}^{\prime }}{F_{m}^{\prime }}$$

The parameters associated with *NPQ* (non-photochemical efficiency: α_NPQ_, and the maximal *NPQ*) were obtained from the tangential model function reported by Eilers and Peeters [46].

### 2.3. Biochemical Indicators

All biochemical determinations were performed on fresh samples of *L. spicata* to preserve the integrity of sensitive compounds such as photosynthetic pigments, phenolic compounds, and antioxidant molecules, which are susceptible to degradation during drying. To enable standardized expression of results and ensure comparability with other studies, all values were converted to dry weight (DW). The fresh–dry weight ratio (FW/DW) was calculated experimentally in this study by drying representative subsamples at 60 °C for 48–72 h. The mean ratio obtained was 2.84, which is consistent with the values previously reported for *L. spicata* under similar environmental conditions [19]. This conversion factor was subsequently used to express biochemical data on a DW basis.

#### 2.3.1. Pigment Content

Photosynthetic pigments, including chlorophyll-*a* (Chl*a*), chlorophyll-*c* (Chl*c*) and total carotenoids (TCs), were extracted from 20 mg of fresh *L. spicata* tissue using 1.5 mL of 90% acetone. Pigment concentrations were determined spectrophotometrically using a spectrophotometer (SPECTROstar Nano, BMG Labtech, Ortenberg, Germany) and calculated according to the method described by Ritchie [47]:

(7) $$Chla\;(mg\;g^{-1}\;DW)=11.47(A_{664}-A_{750})-0.45(A_{630}-A_{750})$$

(8) $$Chlc\;(mg\;g^{-1}\;DW)=22.679(A_{630}-A_{750})-3.404(A_{664}-A_{750})$$

(9) $$TC\left(mg\;g^{-1}\;DW\right)=10(A_{480}-A_{750})$$

where *A* is the absorbance of the extract at a certain wavelength (λ; nm).

#### 2.3.2. Total Phenolic Compounds (PC)

Total phenolic compounds were determined using 0.25 g fresh weight (FW) samples. The samples were pulverized in a mortar using 2.5 mL of 80% methanol. The mixture was transferred to a 15 mL falcon tube and vortexed at 4 °C for 12 to 24 h. Then, it was centrifuged at 4000 rpm for 30 min at 4 °C, and 3 μL of the supernatant was collected for mixing with 237 μL of distilled water and 15 μL of Folin–Ciocalteu reagent [48], as modified by Celis-Plá et al. [42], and shaken vigorously. Finally, 45 μL of 20% anhydrous Na_2_CO_3_ was added. The samples were placed for 2 h in the dark at 4 °C, and the absorbance was measured in a spectrophotometer (SPECTROstar Nano, BMG Labtech, Ortenberg, Germany) at 760 nm. For the calibration curve, phloroglucinol (1,3,5-trihydroxybenzene, Sigma P-3502) was used as a standard.

#### 2.3.3. DPPH Total Antioxidant Capacity

The antioxidant activity of seaweed extracts was estimated indirectly using the method based on the reduction of the stable free radical DPPH (2,2-diphenyl-1-picrylhydrazyl), according to the methodology detailed by Blois [49] and modified by Celis-Plá et al. [42]. The same supernatant used for phenolic compounds was used for DPPH analysis. A total of 150 μL of DPPH prepared in 90% methanol (90MeOH: 10H_2_O) was added to each extract. The reaction was completed after 30 min in the darkness at room temperature (~20 °C), and the absorbance was read at 517 nm in the spectrophotometer (SPECTROstar Nano, BMG Labtech, Ortenberg, Germany). The calibration curve was used to calculate the remaining concentration of DPPH in the reaction mixture after incubation [42].

#### 2.3.4. Quantification of Thiobarbituric Acid Reactive Substances (TBARSs)

TBARSs, as a proxy for lipid peroxidation, were measured according to the methodology of Sáez et al. [50], by spectrophotometry. A total of 200 mg of frozen biomass was triturated with liquid nitrogen; this was then mixed with 300 μL of 0.1% TCA, vortexed for 10 min, and centrifuged at 17,800× *g* for 15 min at 4 °C. Then, 200 μL of the supernatant was taken and mixed with 200 μL of 0.5% Thiobarbituric acid (TBA), and incubated for 45 min at 95 °C. Finally, 200 μL of the supernatant was transferred to a microplate to read the absorbance at 532 nm using a spectrophotometer (SPECTROstar Nano, BMG Labtech, Ortenberg, Germany). Commercial malondialdehyde (MDA, Sigma Aldrich Merck, Darmstadt, Germany) was used for the calibration curve. The data were normalized against total protein concentrations measured by the Bradford method [51].

#### 2.3.5. Total Reactive Oxygen Species (ROSs)

Oxidative damage was measured using the Fluorometric Intracellular Ros Kit (orange) provided by Sigma-Aldrich and modified by Pérez-Hernández et al. [52]. In total, 300 mg of frozen biomass was triturated with liquid nitrogen, and then 300 μL of 0.5 M HCl was added. The samples were shaken vigorously for 10 min at room temperature and then centrifuged for 5 min at 7500 rpm at 4 °C. Subsequently, an aliquot of 100 μL of the supernatant, 100 μL of 100 mM Na_3_PO_4_ buffer (pH 6.8), and 0.5 μL of fluorophore was added to a dark, multicell plate. The relative fluorescence units (RFUs) were measured at 540 nm (excitation) and 570 nm (emission) in a fluorometric spectrophotometer Cytation 5 (BioTek, Agilent, Santa Clara, CA, USA).

### 2.4. Stoichiometry (C:N Ratios)

Total internal C and N contents expressed as % of sample dry weight (DW) were determined by using an element analyzer (model CNHS 932, LECO Corporation, St Joseph, MI, USA) according to Celis-Plá et al. [19].

### 2.5. Total Proteins (TPs), Lipids (TLs), and Carbohydrates (TCs)

TP was measured following the methodology of Slocombe et al. [53], adapted for *L. spicata*. Lyophilized tissue (10 mg) was treated with 0.2 mL of 10% TCA and heated at 95 °C for 15 min. After cooling, 250 µL of distilled water was added, and the sample was centrifuged (10 min, 4400 rpm). The pellet was resuspended in 0.5 mL of Lowry D-NaOH reagent and incubated at 80 °C for 60 min, then centrifuged again. From the supernatant, 50 µL was mixed with 950 µL of the Lowry D-NaOH reagent, incubated for 10 min at room temperature, and followed by 100 µL of 1N Folin reagent and further incubation for 30 min. Absorbance of a 200 µL aliquot was measured at 600 nm (Femto 800 XI, Brazil). Protein concentration was calculated using the method described by Bradford [51], with BSA as standard (Sigma-Aldrich, St. Louis, MO, USA).

TL was extracted from 0.05 g of freeze-dried *L. spicata* using chloroform/methanol (2:1, *v*/*v*) according to the work of Bligh and Dyer [54]. The solvent was evaporated under reduced pressure, and the lipid residue was dried to a constant weight for gravimetric quantification.

TC content was quantified following the work of Albalasmeh et al. [55], adapted for *L. spicata*. A total of 1 mg of the freeze-dried sample was reacted with 3 mL of concentrated H_2_SO_4_, before being vortexed and cooled on ice. Absorbance was measured at 315 nm (Femto 800 XI, São Paulo, Brazil), and concentrations were calculated using a glucose standard curve.

### 2.6. Statistical Analysis

Data normality and homoscedasticity were tested using Shapiro–Wilk and Bartlett tests, respectively. ANOVA was applied to evaluate differences in physiological and biochemical variables across seasons and daily cycles (10:00, 14:00, 18:00 h), according to Underwood [56], using RStudio Team [57] with three replicates per condition. Principal coordinate analysis (PCA) was performed in PRIMER 6 with PERMANOVA+ [58] based on Euclidean distances to assess the contribution of variables to multivariate variance. Arrows in the ordination plot indicate variables with the highest influence. Pearson correlations and linear regressions were calculated in Sigma Plot 15 to explore relationships among dependent variables.

## 3. Results

### 3.1. Environmental Variables

Mean seawater temperatures varied significantly across seasons and time zones (Table 1). Autumn values ranged from 12.88 °C to 13.60 °C, winter from 12.03 °C to 13.42 °C, spring from 12.50 °C to 13.57 °C, and summer from 12.46 °C to 14.98 °C, which was the highest range recorded. The average PAR values showed significant differences in all seasons of the year and between times (Table 1). Irradiance of PAR reached maximal values in spring and summer, i.e., 1323.68 µmol m^−2^ s^−1^ and 1259.68 µmol m^−2^ s^−1^ at 14:00 h, respectively. In contrast, autumn and winter exhibited lower maxima of 989.16 µmol m^−2^ s^−1^ and 375.10 µmol m^−2^ s^−1^, both recorded at 14:00 h (Table 1). UVA radiation values differed significantly throughout winter and spring (Table 1). The highest level was observed in the spring, with values of 11.26 W m^−2^ at 14:00 h. In the summer and autumn, maximum values were recorded at 14:00 h, reaching 9.77 W m^−2^ and 10.01 W m^−2^, respectively.

### 3.2. Photosynthetic Activity

#### 3.2.1. CO_2_ Assimilation

**Figure 2:**
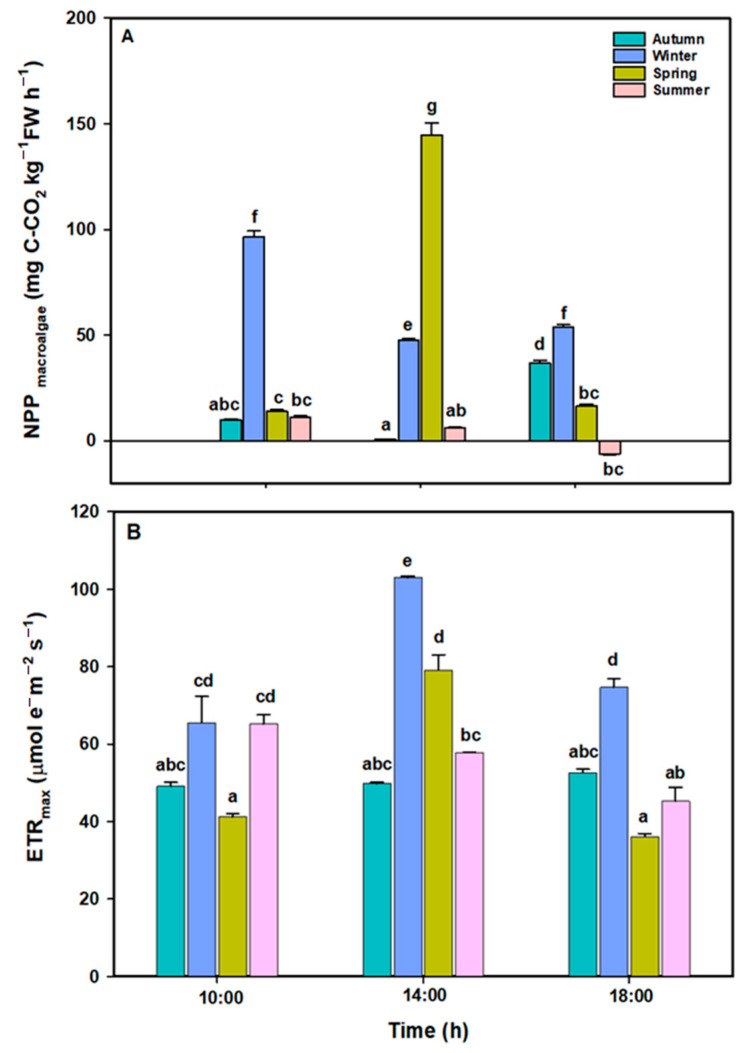
(**A**) NPP_macroalge_ (mg C–CO_2_ kg^−1^ FW h^−1^) and (**B**) maximal electron transport rate (ETR_max_; µmol electrons m^−2^ s^−1^) in *L. spicata* measured in 2022 at Cochoa Beach, Viña del Mar, Chile, during the daily cycle experiments in the autumn, winter, spring, and summer. Letters indicate significant differences after the Tukey test (*p* < 0.01).

The net primary productivity (NPP) of *L. spicata* represents CO_2_ assimilation (mg C–CO_2_ kg^−1^ algae) and was significantly different for all factors (Figure 2A, Table S1). The NPP increased in springtime, with 144.8 mg C–CO_2_ kg^−1^ FW h^−1^ at midday; lower values were recorded in the summertime.

#### 3.2.2. *In Vivo* Chlorophyll *a* Fluorescence

**Figure 3:**
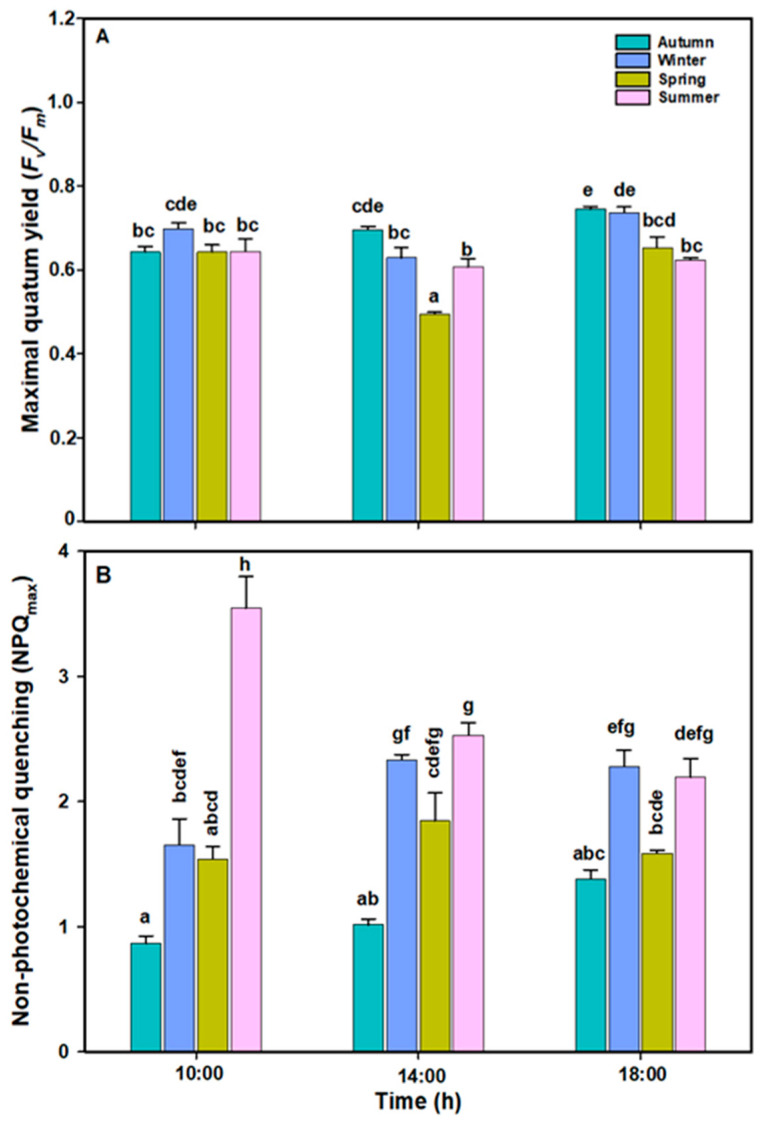
(**A**) Maximal quantum yield (*F_v_*/*F_m_*) and (**B**) non-photochemical quenching (NPQ_max_) in *L. spicata* measured in 2022 at Cochoa Beach, Viña del Mar, Chile, during the daily cycle experiments in the autumn, winter, spring, and summer. Letters indicate significant differences after the Tukey test (*p* < 0.01).

The maximum quantum yield (*F_v_*/*F_m_*) of *L. spicata* showed significant differences across all factors (Figure 3A, Table S1). The *F_v_*/*F_m_* showed the highest mean values (~0.75) at 18:00 h in the autumn and winter. The lowest photo-inhibition, i.e., the lowest *F_v_*/*F_m_* values (~0.50), were observed at midday.

ETR*_max_* as an indicator of maximal photosynthetic activity showed significant differences between all factors (Figure 2B, Table S1). The highest values throughout the year were recorded at midday, with 102.99 and 79.11 µmol electrons m^−2^ s^−1^ in the winter and spring, respectively. In the spring and winter, ETR*_max_* increased from 10:00 to 14:00 and decreased to 18:00 (Figure 2B). In the summertime, the ETR*_max_* values at 10:00 were similar to those in the wintertime; however, in contrast to the winter and the spring, the values decreased throughout the day, reaching the minimal values of 45.26 µmol electrons m^−2^ s^−1^ at 18:00. In the autumn, the ETR*_max_* values did not present differences throughout the day. These findings are consistent with Pearson analyses (Table S2), where a negative correlation was observed between temperature and NPP, and a positive correlation was observed between NPP and ETR_max_, PAR and temperature, and UVA and ETR_max_.

NPQ*_max_* values present significant differences between all factors (Figure 3B, Table S1). In the winter and spring, the NPQ_max_ increased at midday and subsequently decreased by the end of the daily cycle alongside the ETR*_max_*. In contrast, in the summertime, NPQ*_max_* values decreased throughout the day alongside the ETR*_max_*. Maximal NPQ values were observed in summer period at 10:00 (Figure 3B).

### 3.3. Biochemical Responses

**Figure 4:**
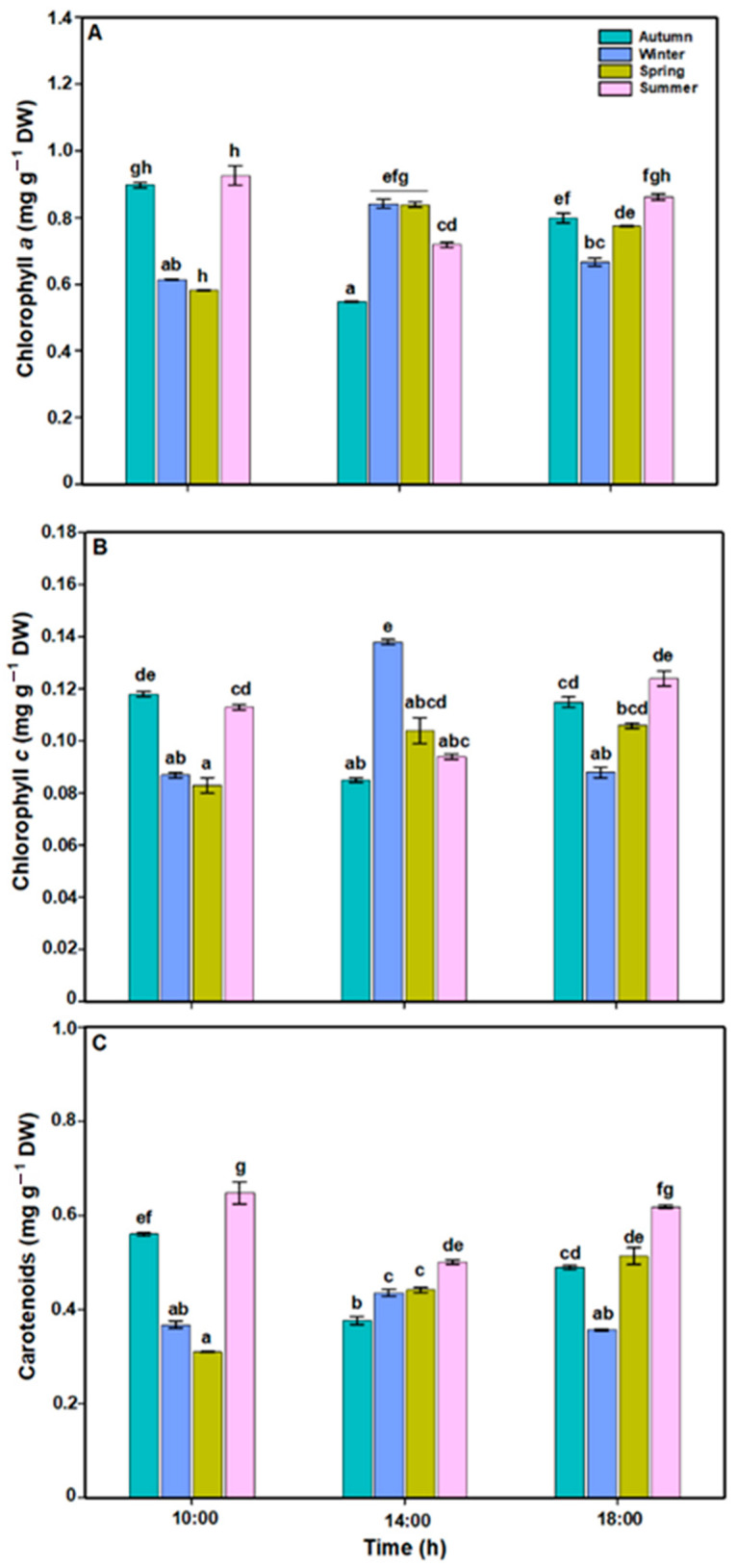
(**A**) Chlorophyll *a* (mg g^−1^ DW), (**B**) chlorophyll *c* (mg g^−1^ DW) and (**C**) carotenoids (mg g^−1^ DW) in *L. spicata* measured in 2022 at Cochoa Beach, Viña del Mar, Chile, during the daily cycle experiments in the autumn, winter, spring, and summer. Letters indicate significant differences after the Tukey test (*p* < 0.01).

The pigment contents in *L. spicata* showed a significant difference across all factors (Figure 4A, B and C, Table S1). Chl*a* and Chl*c* levels increased (~0.9 mg g^−1^ DW, and ~0.14 mg g^−1^ DW, respectively) at midday during the winter and springtime, with lower values in the autumn and summer (~0.5 mg g^−1^ DW, and ~0.09 mg g^−1^ DW, respectively) (Figure 4). Total carotenoid (TC) values were always highest during the summer (~0.65 mg g^−1^ DW), while the lowest values (~0.30 mg g^−1^ DW) were in the winter and spring (Figure 4C).

**Figure 5:**
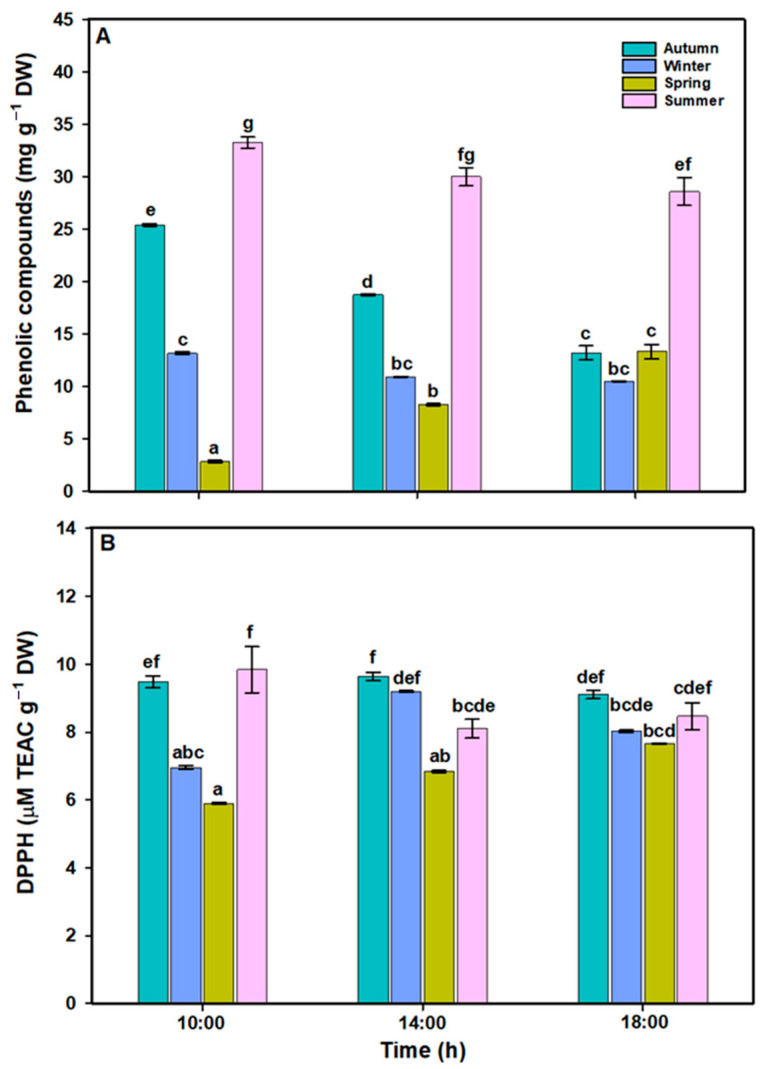
(**A**) Phenolic compounds (mg g^−1^ DW) and (**B**) DPPH (µM TEAC g^−1^ DW) in *L. spicata* measured in 2022 at Cochoa Beach, Viña del Mar, Chile, during the daily cycle experiments in the autumn, winter, spring, and summer. Letters indicate significant differences after the Tukey test (*p* < 0.01).

Phenolic compounds (PCs) in *L. spicata* showed significant differences across all factors (Figure 5A, Table S1). The PC values were higher in the summer (~33.29 mg g^−1^ DW) and autumn (~25.44 mg g^−1^ DW). PC observed a similar pattern in the winter, with a maximum value ~ 13.18 mg g^−1^ DW at 10:00. In contrast, PC values increased throughout the day in the springtime, reaching a maximum of ~14 mg g^−1^ DW at 18:00 h. The DPPH content in *L. spicata* exhibited significant differences across all factors (Figure 5B, Table S1). During the daily cycle, DPPH was higher in the spring (~6–8 µM TE mg^−1^ DW) and wintertime (~9 µM TE mg^−1^ DW), whereas in the summer, DPPH showed a lower value (~8 µM TE mg^−1^ DW). These results are consistent with the positive Pearson correlation (Table S2) observed between PCs and DPPH.

**Figure 6:**
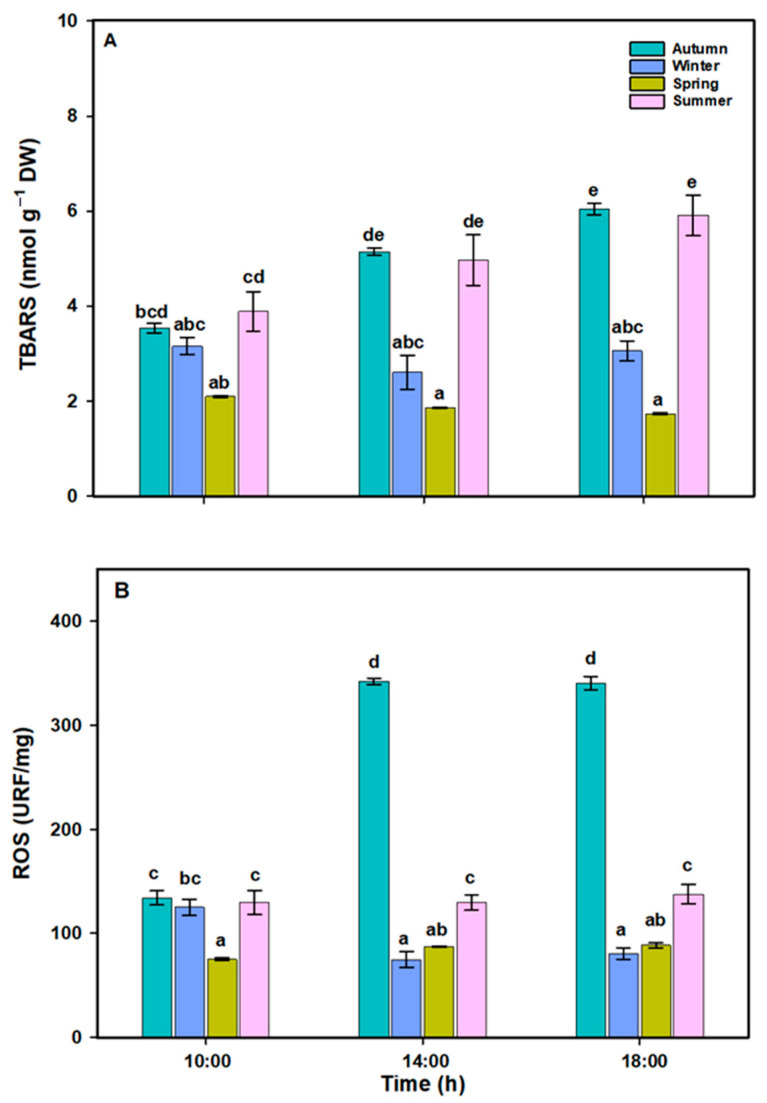
(**A**) Thiobarbituric acid reactive substances (TBARSs) (nmol g^−1^ DW) and (**B**) total reactive oxygen species (ROSs) in *L. spicata* exposed to daily cycle experiments in the autumn, winter, spring, and summer of 2022 at Cochoa Beach, Viña del Mar, Chile. Letters indicate significant differences after the Tukey test (*p* < 0.01).

The TBARS content in *L. spicata* exhibited a significant difference across all factors (Figure 6A, Table S1). During the autumn and summer, higher values were observed throughout the daily cycle, between ~6.04 and 5.91 nmol g^−1^ DW, respectively. In contrast, the TBARS content decreased in the spring, ranging from ~2 to 1.73 nmol g^−1^ DW. The total ROS content in *L. spicata* showed significant differences across all factors (Figure 6B, Table S1). The highest values, reaching 342.45 RFU/mg, were observed in the autumn at 14:00 and 18:00 h; in contrast, the lowest values, around 75.16 RFU/mg, were recorded in the winter and spring at the same times.

### 3.4. Stoichiometry (C:N)

**Figure 7:**
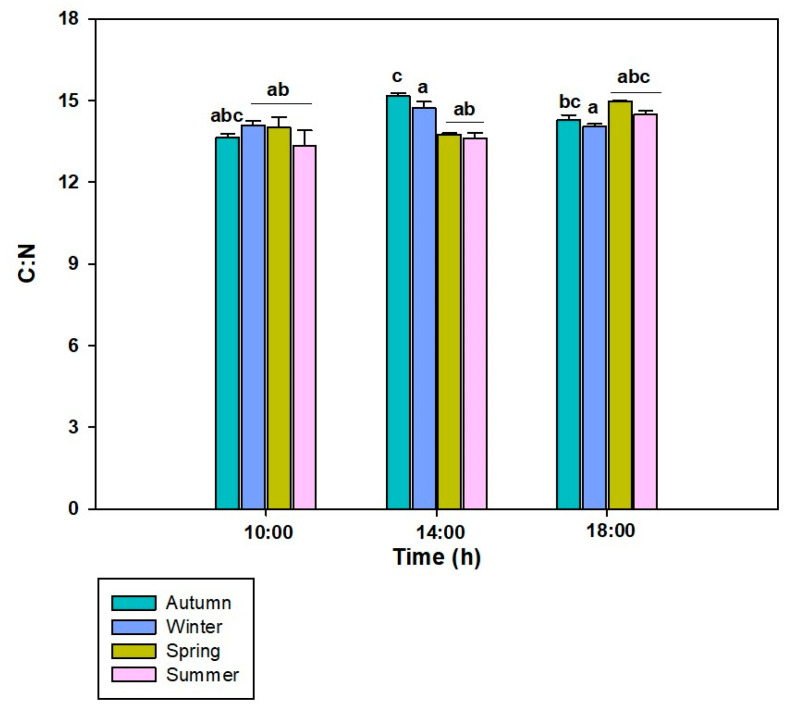
C:N ratio to *L. spicata* measured in 2022 at Cochoa Beach, Viña del Mar, Chile, during the daily cycle experiments in the autumn, winter, spring, and summer. Letters indicate significant differences after the Tukey test (*p* < 0.01).

Regarding the C:N ratio in *L. spicata*, significant differences were observed across all factors (Figure 7, Table S1). Overall, the C:N ratio showed slight variations throughout the day, reaching its highest value in the autumn at 14:00 h (~15.16 mgC g^−1^ DW/ mgN g^−1^ DW). In contrast, summer exhibited consistently lower ~13.35 mgC g^−1^ DW/ mgN g^−1^ DW.

### 3.5. Total Proteins, Lipids and Carbohydrates

**Figure 8:**
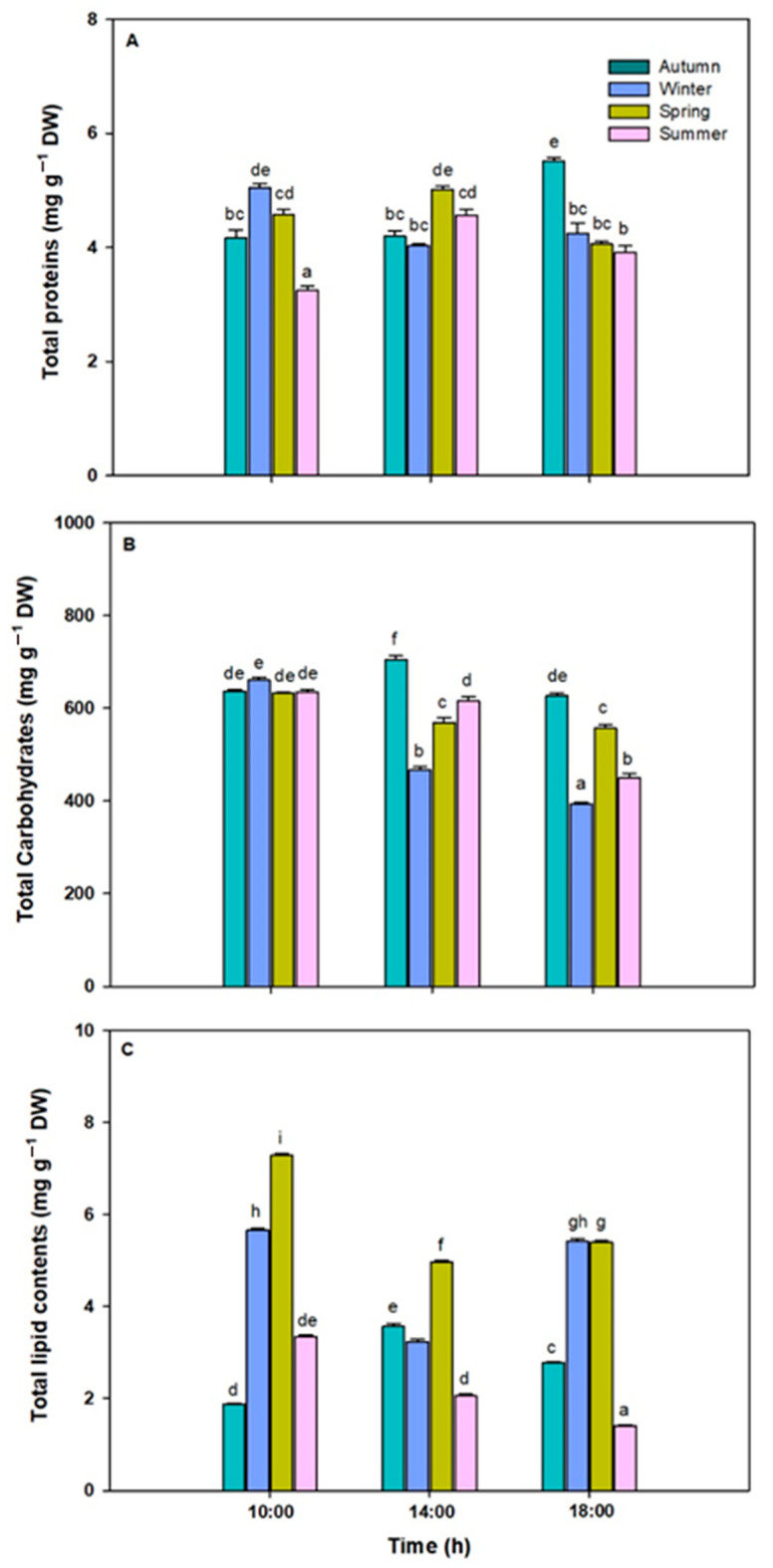
(**A**) Total protein (mg g^−1^ DW), (**B**) total carbohydrate (mg g^−1^ DW), and (**C**) total lipid contents in *L. spicata* exposed to daily cycle experiments in the autumn, winter, spring, and summer times. Letters indicate significant differences after the Tukey test (*p* < 0.01).

TP, TC, and TL contents in *L. spicata* showed significant differences across all factors (Figure 8A–C, Table S1). TP content was higher in the autumn, at ~5.52 mg g^−1^ DW at the end of the experimental time (Figure 8A). Indeed, the TL content was higher in ~7.29 mg g^−1^ DW at the start of the experimental period (Figure 8B). Finally, TC content was higher in the autumn time during the central hours of the day, at ~ 705.12 mg g^−1^ DW (Figure 8C).

### 3.6. Multivariable Results

**Figure 9:**
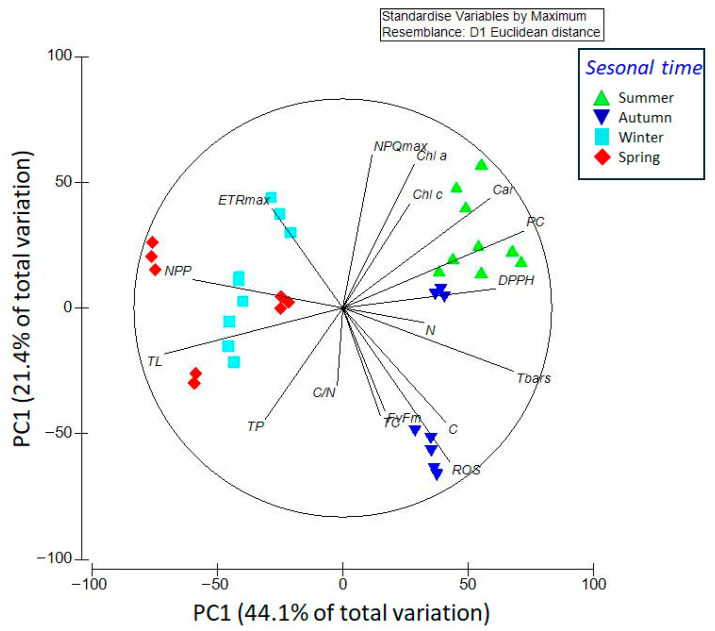
Principal components diagram based on 17 ecophysiological variables (NPP, ETR*_max_*, F*_v_*/F*_m_*, NPQ*_max_*, C, N, C/N, Chl*a*, Chl*c*, Car, PCs, DPPH, TBARSs, TCs, TPs, TLs, and ROS) and treatment data from *Lessonia spicata* experiments.

Principal component analysis (PCA) revealed distinct seasonal patterns in the multivariate structure of the physiological and biochemical profiles of *Lessonia spicata* (Figure 9). The first two principal components explained 65.5% of the total variance (PC1: 44.1%, PC2: 21.4%). Samples were clearly separated by season within the ordination space, reflecting strong covariation among key functional traits.

Spring and winter samples clustered along vectors associated with elevated net primary productivity (NPP) and total lipid content (TL), indicative of metabolically active growth states. Spring samples were further associated with high total protein (TP) and moderate to high electron transport rates (ETR_max_), suggesting an optimal physiological condition characterized by efficient carbon assimilation and macronutrient biosynthesis under favorable conditions of moderate temperature and high irradiance. Winter samples aligned with elevated ETR_max_ and moderate non-photochemical quenching (NPQ_max_), reflecting high photosynthetic efficiency during this season.

In contrast, summer samples were primarily associated with elevated pigment concentrations (Chl*a*, Chl*c*, Car) and antioxidant activity (DPPH, PC), indicating enhanced photoprotective responses to high irradiance and thermal stress. Autumn samples clustered with variables linked to oxidative stress and photo-inhibition, including reactive oxygen species (ROSs), maximum quantum yield (*F_v_*/*F_m_*), and total carbon (C), suggesting a distinct stress-related physiological state. These multivariate patterns, shaped by the covariance among ecophysiological variables, offer integrative insights into the seasonal dynamics of *L. spicata* that would not be apparent through univariate analyses alone.

## 4. Discussion

This study demonstrates a clear seasonal modulation of *L. spicata* in its physiological, biochemical, and photochemical traits, reflecting its capacity to adjust metabolic functions in response to fluctuating environmental conditions. The integration of multivariate analyses provides a coherent understanding of how environmental drivers such as temperature, solar irradiance (PAR and UVA), and seasonal photoperiods interact to shape the organism’s performance across all seasons. Winter and spring were consistently associated with enhanced carbon assimilation and productivity, as evidenced by elevated net primary productivity (NPP), maximal electron transport rate (ETR*_max_*), total lipids (TLs), and total protein (TP) content. These variables are clustered together in multivariable analyses in combination with PCA and aligned with periods of moderate temperature and high irradiance. Spring emerged as a physiological optimum for *Lessonia spicata*, where favorable environmental conditions promoted simultaneous enhancement of photosynthetic performance and macronutrient biosynthesis. This was supported by strong positive correlations among net primary productivity (NPP), electron transport rate (ETRmax), total protein (TP), and total lipids (TLs), indicating a metabolically active and efficient physiological state. In contrast, samples collected during the summer and autumn exhibited distinct physiological adjustments associated with environmental stress. During the summer, although irradiance remained high, elevated seawater temperatures appeared to suppress photosynthetic efficiency and metabolic activity, as reflected by reduced NPP, ETRmax, and TL values. Nevertheless, concentrations of photosynthetic pigments (Chl *a*, Chl *c*, and total carotenoids) and antioxidant compounds (DPPH and phenolic compounds, PC) were highest during this season. These responses suggest that *L. spicata* activates photoprotective and antioxidant mechanisms to cope with excessive light and thermal stress, which is consistent with previous observations [19,36].

In autumn, the physiological profile of *L. spicata* was characterized by elevated levels of reactive oxygen species (ROSs) and a decline in the maximum quantum yield of photosystem II (*F_v_*/*F_m_*), indicating the occurrence of oxidative stress and photoinhibition. Concurrently, the accumulation of total carbon and increased C:N ratios suggest a metabolic shift toward carbon storage, potentially at the expense of growth and protein synthesis.

These seasonal contrasts underscore the species’ capacity for flexible, seasonally adjusted metabolic strategies, allowing *L. spicata* to maintain functional performance across a dynamic and often stressful coastal environment. In previous studies, the seasonal plasticity and photoprotective strategies of *L. spicata* have been described in response to temperature and irradiance variability [19,35,36]. This is the first study to quantify its direct CO_2_ assimilation rate and assess the seasonal dynamics of key macronutrients such as proteins, lipids, and carbohydrates in this species from the Bay of Valparaíso. Due to their high NPP, macroalgae are increasingly recognized as significant contributors to coastal carbon sequestration [59,60,61,62]. Duarte et al. [62] estimated an average annual PPN of ~0.59 kg C m^−2^ yr^−1^ (equivalent to 161.64 mg C m^−2^ day^−1^) in intertidal kelp forests, such as *Durvillaea antarctica*. Similarly, Eger et al. [63] reported that kelp forests dominated by species such as *Ecklonia* spp., *Lessonia* spp., *Laminaria* spp., *Saccharina* spp., *Macrocystis* spp., and *Nereocystis* spp. can sequester between 31 and 214 g C m^−2^ yr^−1^, with a carbon assimilation rate close to 10% of their NPP. However, the seasonal dynamics of NPP in intertidal macroalgae remain poorly understood, despite clear evidence that environmental factors, particularly temperature and photosynthetically active radiation (PAR), drive strong seasonal variation in productivity [28,64,65]. The physiological and biochemical responses of brown macroalgae, including carbon allocation and photoprotection, are also modulated by temperature, UV, and PAR [19,66,67,68].

In our study, the highest values for NPP (~144.8 mg C–CO_2_ kg^−1^ FW h^−1^) and ETR*_max_* (~102.9 µmol e- m^−2^ s^−1^) were recorded in the spring at midday, under high PAR (~1323.6 µmol photons m^−2^ s^−1^), moderate UVA (~11.26 W m^−2^), and seawater temperature conditions (~13.6 °C). These conditions were associated with the enhanced biosynthesis of organic compounds such as TPs (~5.013 mg g^−1^ DW) and TLs (~4.973 mg g^−1^ DW). In contrast, during the summer, at midday, although irradiance levels (PAR and UVA) were similar to those recorded in the spring, lower NPP (~6.034 mg CC–CO_2_ kg^−1^ FW h^−1^) and ETR*_max_* (~57.81 µmol e^-^ m^−2^ s^−1^), and TL (~2.065 mg g^−1^ DW) values were recorded, coinciding with the water temperatures exceeding 14 °C. This suggest that thermal stress may impair photosynthetic efficiency, carbon fixation, and energy storage in *L. spicata*, as has been observed in other macroalgae assemblages where rising temperatures increase respiration rates over photosynthetic rates, which may reduce NPP and thus carbon sequestration capacity [69,70,71].

Interestingly, although UVA radiation is typically harmful to photosynthetic organisms, it did not appear to significantly limit the photosynthetic capacity of *L. spicata* in the months of highest productivity. In fact, UVA-Blue photoreceptors have been shown to activate enzymes involved in carbon assimilation, such as carbonic anhydrase, nitrogen metabolism, and nitrate reductase, suggesting a potential regulatory role of UVA under moderate exposure [72,73,74]. This dual role of UVA as both a stressor and facilitator depend on its intensity and duration of exposure [75]. For comparison, Migné et al. [76] reported gross primary productivity values for *L. digitata* ranging from ~24 to 456 mg C m^−2^ h^−1^, and ETR*_max_* ~50–180 µmol electrons m^−2^ s^−1^ during spring and summer in the North Atlantic (France), under high PAR levels of ~1933-2248 µmol photons m^−2^ s^−1^ and seawater temperatures between ~11–18 °C. Although the irradiance conditions in our study were comparable, *L. spicata* showed a marked reduction in NPP and TLs during summer, coinciding with temperatures exceeding 14 °C. This contrast suggests that elevated temperatures may constrain carbon fixation in *L. spicata*, despite favorable light conditions. These findings reinforce the notion that macroalgal productivity is not solely dependent on irradiance, but rather on the complex interaction of multiple environmental drivers, including temperature.

Variations in environmental stressors influence not only NPP and ETR*_max_*, but also key metabolic process such as the Calvin cycle, growth, reproduction, metabolic rates, biochemical composition, and overall distribution of macroalgae [77,78,79]. In our study, the highest values of the C:N ratio (~15) were recorded during the autumn at midday, when NPP and ETR*_max_* were lowest. However, this coincided with elevated levels of TL and carbohydrate content, suggesting that *L. spicata* may have experienced nitrogen limitation and redirected assimilated carbon toward the synthesis of energy storage compounds rather than rapid growth (e.g., protein synthesis). In contrast, a lower C:N ratio (~13.5) was observed during the summer at midday, along with an increase in TPs; this likely reflects enhanced protein and enzyme production linked to higher nitrogen availability, as described by Twigg et al. [80]. TLs in *L. spicata* ranged from ~1.41 to 7.29 mg g^−1^ DW, which are comparable to the values reported by Meng et al. [81] for *Ascophyllum nodosum* (brown alga), ranging between 0.35 and 4.72 mg g^−1^ DW. Regarding TPs, *L. spicata* showed relatively low average concentrations (~4.38 mg g^−1^ DW), which are consistent with the findings of Smale et al. [64], who pointed out that light intensity can act as a determining factor in biomass accumulation and storage due to its positive correlation with NPP. In addition to the effect of temperature effects on carbon sequestration discussed above, these results indicate that seasonal variability, particularly thermal stress, may influence not only the rate but also the quality of carbon fixed by *L. spicata*, potentially affecting its ecological performance, biogeographic distribution, and long-term ecosystem-climate services. Nevertheless, *L. spicata* has been shown to adjust its physiological processes in response to environmental stressors over daily and seasonal cycles through a mechanism known as photoacclimation [19]. This strategy allows macroalgae to optimize their photosynthetic performance by adapting to variations in irradiance and spectral light conditions [82]. Consequently, it implies a reduction in the *F_v_*/*F_m_* ratio, and an increase in the production of reactive oxygen species (ROSs) and lipid peroxidation markers such as TBARSs (oxidants). To mitigate these effects, macroalgae also activate photoprotective mechanisms, including the production of PCs and carotenoids, and the dissipation of excess energy as heat through NPQ*_max_* [19,79,83]. In this sense, our results show that *L. spicata*, in the springtime, at midday, recorded a greater decrease in the *F_v_*/*F_m_* ratio and an increase in TBARS levels compared to the rest of the seasons in the year, indicating a greater photosystem II photoinhibition [84] and an increase in oxidative damage at the membrane level. This stress was accompanied by a significant increase in NPQ*_max_* as well as in the contents of Chl*a*, Chl*c*, carotenoids, ROSs, PCs, and DPPH. The increase in ROS production could have been driven by the high irradiance, which caused an overexcitation of the photosynthetic apparatus and the activation of multiple protective mechanisms, which is reflected in the increase of NPQ*_max_* [85]. Together with the accumulation of Chl*a*, accessory pigments and PCs, which are associated with photoprotection and free radical neutralization, reflect an integrated defense strategy against environmental conditions [42,85]. In contrast, during the summer at midday, although a reduction in the F*_v_*/F*_m_* ratio was observed, NPQ*_max_*, Chl*a*, Chl*c*, carotenoid, PC, DPPH, and TBARS values decreased significantly. However, no relevant changes were detected in the total concentration of ROSs. These results suggest that *L. spicata*, under conditions of higher temperature and light stress, reduces or suppresses metabolic reactions associated with photosynthesis and photoprotection mechanisms, which is reflected in the reduction in photosynthetic pigments, and lower ETR_max_, NPP, and NPQ_max_. This could demonstrate that *L. spicata* presents a greater physiological vulnerability in the summer with implications for its ecological performance and resilience under climate change scenarios (higher irradiance and temperature). These findings agree with those reported by Figueroa et al. [44], who demonstrated that prolonged exposure to UV radiation significantly reduces the content of photosynthetic pigments, particularly Chl*a*, in *Ulva rotundata*, associating this effect with photooxidative damage and the inhibition of pigment biosynthesis.

In contrast, the ETR_max_ values for *L. spicata* reported by Celis-Plá et al. [19] (100–120 μmol m^−2^ s^−1^) and Zúñiga et al. [36] (60–100 μmol m^−2^ s^−1^) during the summer were notably higher than those observed in the present study (40–65 μmol m^−2^ s^−1^). These differences may be attributed to variations in environmental conditions, such as elevated seawater temperatures or the cumulative effects of prior stress exposure. Additionally, it is important to highlight that our measurements were taken at later times of day compared to those in the referenced studies. As a result, the combined impact of peak irradiance and thermal load may have exceeded the photoprotective capacity of *L. spicata*, leading to more pronounced photoinhibition.

A similar trend was reported by Figueroa et al. [45], who documented a concurrent decline in Fv/Fm and ETRmax in *Ulva rigida* following medium-term (7-day) exposure to UVB radiation, demonstrating the cumulative effects of high irradiance on the functional integrity of the photosynthetic apparatus. Taken together, these findings suggest that under the combined influence of elevated temperatures and intense solar radiation—conditions characteristic of summer—the photoacclimation capacity of *L. spicata* may become compromised, ultimately impairing its photosynthetic efficiency and carbon assimilation potential.

## 5. Conclusions

This study provides novel evidence of the seasonal modulation of CO_2_ assimilation, productivity, and macronutrient biosynthesis in *Lessonia spicata*, underscoring its physiological plasticity in response to fluctuating environmental conditions. Spring emerged as the optimal season for photosynthetic efficiency and carbon storage, with elevated values of NPP, ETR_max_, total proteins (TPs), and total lipids (TLs), driven by moderate seawater temperatures and high irradiance levels.

In contrast, the summer of 2022 was marked by extreme environmental conditions—high irradiance combined with elevated water temperatures—that negatively impacted photosynthetic performance and macronutrient synthesis. These findings suggest that simultaneous exposure to thermal and light stress may constrain the carbon sequestration potential of *L. spicata*.

Although previous studies have described the photoprotective strategies of this species, our results indicate that its photoacclimation capacity may be seasonally limited, especially under compounding environmental stressors. Moreover, the direct quantification of CO_2_ assimilation, combined with seasonal trends in macronutrient content, highlights the ecological significance of *L. spicata* not only as a key primary producer in intertidal ecosystems, but also as a promising nature-based solution for climate change mitigation in coastal environments.

## Figures and Tables

**Table 1:** Abiotic parameters: temperature (°C), pH, salinity, photosynthetically active radiation (PAR; µmol photons m^−2^ s^−1^), and ultraviolet-A radiation (UVA; W m^−2^) at Cochoa Beach in Valparaíso Bay during the daily cycle in the autumn, winter, spring, and summer (2022). Letters indicate significant differences after the Tukey test (*p* < 0.01).

Season	Dayle Cycle	Temperature (°C)	pH	Salinity	PAR (µmol m^−2^ s^−1^)	UVA (W m^−2^)
Autumn	10:00	12.9 ± 0.1 ^c^	7.9 ± 0.1	34.4 ± 0.1	335.37 ± 8.99 ^ab^	4.77 ± 0.09 ^a^
	14:00	13.6 ± 0.1 ^e^	8.1 ± 0.1	34.4 ± 0.1	989.16 ± 19.04 ^cd^	10.0 ± 0.1 ^a^
	18:00	13.2 ± 0.1 ^d^	8.1 ± 0.1	34.3 ± 0.1	332.58 ± 14.48 ^bc^	4.54 ± 0.21 ^a^
Winter	10:00	12.0 ± 0.1 ^c^	7.8 ± 0.1	34.5 ± 0.1	269.12 ± 8.63 ^ab^	4.66 ± 0.10 ^c^
	14:00	13.2 ± 0.1 ^d^	8.0 ± 0.1	34.7 ± 0.1	375.10 ± 11.93 ^ab^	5.61 ± 0.13 ^d^
	18:00	13.4 ± 0.1 ^de^	8.1 ± 0.1	34.7 ± 0.1	0.24 ± 0.04 ^a^	4.25 ± 0.21 ^a^
Spring	10:00	12.5 ± 0.1 ^b^	7.7 ± 0.1	36.0 ± 0.1	457.01 ± 16.48 ^ab^	2.70 ± 0.08 ^b^
	14:00	13.6 ± 0.1 ^e^	8.4 ± 0.1	35.6 ± 0.1	1323.7 ± 23.3 ^d^	11.3 ± 1.7 ^a^
	18:00	13.4 ± 0.1 ^de^	8.2 ± 0.1	36.3 ± 0.1	228.56 ± 6.39 ^ab^	4.21 ± 0.07 ^a^
Summer	10:00	12.5 ± 0.1 ^b^	7.8 ± 0.1	34.9 ± 0.1	224.26 ± 12.44 ^ab^	2.70 ± 0.14 ^a^
	14:00	14.3 ± 0.1 ^f^	8.0 ± 0.1	34.9 ± 0.1	1259.7 ± 30.8 ^d^	9.77 ± 0.09 ^a^
	18:00	14.9 ± 0.1 ^g^	8.2 ± 0.1	34.9 ± 0.1	134.28 ± 12.05 ^cd^	1.99 ± 0.02 ^a^

## Data Availability

Data is contained within the article and Supplementary Materials.

## Supplementary Materials

The following supporting information can be downloaded at https://www.mdpi.com/article/10.3390/plants14152341/s1: Table S1: ANOVA results; Table S2: Pearson correlation.
