# Supplementary material for: Environmental Stressors Modulating Seasonal and Daily Carbon Dioxide Assimilation and Productivity in Lessonia spicata
Source: Plants (Basel). 2025 Jul 29;14(15):2341. doi: 10.3390/plants14152341 (PMC12348734; doi:10.3390/plants14152341)
Supplement: Supplementary file 1 [file plants-14-02341-s001.zip › plants-3727038-supplementary.pdf]

SUPPLEMENTARY MATERIAL

Table S1. ANOVA results for ecophysiological responses in *Lessonia spicata* ($p < 0.05$:*; $p < 0.01$: **; $p < 0.001$:***). Results considered were F_v/F_m, ETR_{max}, Chlorophyll *a*, Chlorophyll *c*, Carotenoids, C:N, Phenolic Compounds, DPPH, TBARS, ROS and NPP, under daily cycle experiments during Autumn, Winter, Spring, and Summer during 2022 in Valparaíso Bay.

		Lessonia spicata			
		df	***MS***	***F***	***P***
**F_v/F_m**	*Season (s)*	3	**0.021**	**22.740**	***
	Hour (h)	2	**0.021**	**23.118**	***
	*s*h*	6	**0.007739**	**7.441**	***
	Res	24	0.0009		
**ETR_{max}**	*Season (s)*	3	**1829.300**	**83.200**	***
	Hour (h)	2	1426.600	64.890	
	*s*h*	6	**561.500**	**25.540**	***
	Res	24	22.000		
**NPQ_{max}**	*Season (s)*	3	**4.463**	**77.181**	***
	Hour (h)	2	**0.015**	**0.258**	***
	*s*h*	6	**0.730**	**12.621**	***
	Res	24	0.058		
**Chlorophyll *a***	*Season (s)*	3	**0.0279**	**69.06**	***
	Hour (h)	2	**0.0044**	**10.88**	***
	*s*h*	6	**0.0745**	**184.79**	***
	Res	24	0.0004		
**Chlorophyll *c***	*Season (s)*	3	**0.0002**	**6.661**	**
	hour (h)	2	**0.0002**	**5.252**	*
	*s*h*	6	**0.0015**	**38.968**	***
	Res	24	0.002		
**Carotenoids**	*Season (s)*	3	**0.0709**	**253.36**	***
	hour (h)	2	**0.0094**	**33.54**	***
	*s*h*	6	**0.0240**	**85.73**	***
	Res	24	0.002		
**C:N**	*Season (s)*	3	**0.5453**	**3.151**	*
	hour (h)	2	**1.5209**	**8.787**	**
	*s*h*	6	**0.9926**	**5.735**	***
	Res	24	0.1731		
**Phenolic Compounds**	*Season (s)*	3	**895.100**	**960.790**	***
	Hour (h)	2	**16.900**	**18.130**	***
	*s*h*	6	**67.900**	**72.590**	***

	Res	24	0.900		
**DPPH**	*Season (s)*	3	**11.385**	**59.274**	***
	Hour (h)	2	0.510	2.656	
	*s*h*	6	**2.780**	**14.473**	***
	Res	24	0.192		
**TBARS**	*Season (s)*	3	**20.349**	**93.457**	***
	Hour (h)	2	**3.108**	**14.276**	***
	*s*h*	6	**1.728**	**7.934**	***
	Res	24	0.218		
**ROS**	*Season (s)*	3	**683303**	**520.63**	***
	Hour (h)	2	7823	59.63	
	*s*h*	6	**12560**	**95.74**	***
	Res	24	131		
**NPP**	*Season (s)*	3	**4512**	**827.1**	***
	Hour (h)	2	**1376**	**252.3**	***
	*s*h*	6	**750**	**137.5**	***
	Res	24	5		
**Proteins**	*Season (s)*	3	**0.9499**	**33.724**	***
	Hour (h)	2	**0.1373**	**4.876**	*
	*s*h*	6	**1.5010**	**53.287**	***
	Res	24	0.0282		
**Lipids**	*Season (s)*	3	**26.059**	**4971.3**	***
	Hour (h)	2	**3.748**	**715.9**	***
	*s*h*	6	**3.750**	**715.5**	***
	Res	24	0.005		
**Carbohydrates**	*Season (s)*	3	**34189**	**219.69**	***
	Hour (h)	2	**353.58**	**353.58**	***
	*s*h*	6	**14607**	**93.86**	***
	Res	24	156		

Res: Residual

Table S2. Pearson correlation of the data set, respect to Chla, Chlc, total carotenoids (TC), DPPH, Phenolics compounds (PC), Tbars, %N, %C, C:N, ROS, ETR_{max}, F_v/F_m, NPQ_{max}, Ek_{NPQ}, NPP, Proteins, Lipids, Carbohidrates (CH), Temperature (T), pH, Salinity, Photosynthetically active radiation (PAR), and Ultraviolet-A radiation (UVA). **correlation is significant at the $p<0.05$

Variables	*Chlc*	*Car*	*DPPH*	*PC*	*Tbars*	*N*	*C*	*C:N*	*ROS*	ETR_{max}	F_v/F_m	NPQ_{max}	*NPP*	*TP*	*TL*	*TC*	*T°C*	*pH*	*Salinity*	*PAR*	*UVA*
Chla	**0.8	**0.83	**0.42	**0.46	0.05	0.16	-0.11	-0.26	-0.22	0.21	-0.36	0.37	0.01	-0.32	**-0.57	-0.27	0.17	0.31	-0.05	-0.22	-0.22
Chlc		**0.60	**0.47	0.27	0.12	-0.07	-0.06	0.06	-0.09	0.32	-0.17	0.20	-0.07	-0.27	**-0.58	-0.36	0.22	0.27	-0.15	-0.29	-0.26
TC			**0.54	**0.80	**0.37	0.30	0.17	-0.20	0.01	-0.16	-0.21	**0.397	**-0.35	**-0.46	**-0.71	-0.05	**0.32	0.17	-0.09	-0.19	-0.27
DPPH				**0.61	**0.51	0.22	**0.35	0.06	**0.47	0.11	0.23	0.15	**-0.43	**-0.41	**-0.71	0.04	0.15	0.06	**-0.60	-0.11	-0.01
PC					**0.57	**0.42	0.25	-0.27	0.16	-0.19	-0.06	**0.40	**-0.53	**-0.52	**-0.77	0.13	**0.34	-0.08	**-0.36	0.03	-0.07
Tbars						0.28	**0.48	0.07	**0.7	-0.24	0.37	0.01	**-0.46	0.05	**-0.71	0.13	**0.46	0.02	**-0.62	**0.03	**-0.01
N							**0.56	**-0.69	0.29	-0.27	-0.22	-0.01	-0.15	0.00	-0.31	**0.417	0.05	-0.03	-0.14	0.30	0.16
C								0.20	**0.71	**-0.51	0.05	**-0.43	**-0.338	0.04	-0.34	0.46	0.33	0.25	-0.09	0.35	0.30
C:N									0.26	-0.11	0.31	**-0.35	-0.13	0.01	0.06	-0.10	0.22	0.24	0.09	-0.07	0.06
ROS										-0.29	**0.43	**-0.38	-0.25	0.29	-0.36	0.50	0.11	0.07	-0.52	0.19	0.26
ETR_{max}											-0.19	**0.41	**0.56	0.01	-0.01	-0.39	-0.12	0.15	-0.30	0.07	0.24
F_v/F_m												-0.18	-0.36	0.08	0.06	0.03	-0.24	**-0.36	**-0.43	**-0.51	**-0.39
NPQ_{max}													-0.02	**-0.47	-0.12	**-0.349	0.08	-0.10	0.03	-0.11	-0.21
NPP														0.52	**0.39	-0.13	-0.20	**0.41	0.07	0.23	**0.42
TP															0.22	0.24	-0.11	0.14	-0.11	0.27	**0.34
TL																0.05	**-0.55	-0.24	**0.55	-0.08	-0.08
TC																	**-0.44	**-0.42	-0.09	**0.41	0.30
T°C																		**0.66	0.03	0.28	0.25
pH																			0.14	0.29	**0.46
Salinity																				0.10	-0.08
PAR																					**0.91
